# Relevance of cortisol and copeptin blood concentration changes in an experimental pain model

**DOI:** 10.1038/s41598-022-08657-4

**Published:** 2022-03-19

**Authors:** Claudine A. Blum, Laëtitia Velly, Christine Brochet, Frédéric Ziegler, Marie-Pierre Tavolacci, Pierre Hausfater, Virginie Eve Lvovschi

**Affiliations:** 1 Service d’Accueil des Urgences, GH Pitié-Salpêtrière, Assistance Publique Hôpitaux de Paris, 47-83 Boulevard de l’Hôpital, 75651 Paris, France; 2 Medical University Clinic, Kantonsspital Aarau, Tellstrasse 25, Haus 7, 5001 Aarau, Switzerland; 3 Sorbonnes Universités, Faculté de Médecine, GRC-14 BIOSFAST, Paris, France; 4 Service de Biochimie Endocrinienne et Oncologique, GH Pitié-Salpêtrière, Assistance Publique Hôpitaux de Paris, 47-83 Boulevard de l’Hôpital, 75651 Paris, France; 5 Biochemistry Department, Hospital Charles Nicolle, Rouen University, 76000 Rouen, France; 6 Digestive Tract Environment and Nutrition, INSERM U1073, Normandie University, 76000 Rouen, France; 7 UNIROUEN, INSERM U1073, INSERM CIC-CRB 1404, Rouen University Hospital, Normandie University, 76000 Rouen, France; 8 Emergency Department, Rouen University Hospital, 76031 Rouen, France

**Keywords:** Biomarkers, Experimental models of disease, Clinical trial design, Pain, Diagnostic markers

## Abstract

The effect of pain and analgesics on stress biomarkers is not well studied. We evaluated the effect of acute pain and analgesics on serum cortisol and copeptin in an experimental pain model in healthy volunteers. Healthy volunteers presented at 8 a.m. for an experimental pain stimulation. Cortisol and copeptin levels were measured before, during and after electrophysiological stimulation, first before and then during opioid delivery. Difference in biomarker levels compared to baseline levels was calculated, and potential influencing factors were evaluated by linear regression analysis. Cortisol decreased by 13% during the 10 min of rest at baseline, but copeptin did not change significantly. Cortisol had a median decrease of −24% or −83 nmol/l (−44 to −124 nmol/l, p = 0.0002) during the electrophysiological stimulation training session, while the median difference for copeptin was −22% or −1.01 pmol/l (−2.35 to 0.08 pmol/l, p = 0.0003). After administration of opioids, cortisol did not decrease but increased by 3% (p = 0.043), indicating an increasing opioids effect on cortisol. This effect was not visible for copeptin (median change −0.003 pmol/l (−0.50 to 0.24), p = 0.45). In this experimental pain model performed in the morning, moderate pain did not have a relevant effect on cortisol or copeptin levels, whereas opioids led to a discrete peak of cortisol.

Clinicaltrials.gov identifier: NCT01975753 (registered on November 5, 2013, before start of recruitment).

## Introduction

Pain is the most common reason for presentation to the emergency department (ED)^1,2^, but its treatment is often not adequately addressed^2–4^. If not treated early and adequately, acute pain can lead to prolonged hospitalization through slower healing and may transform to chronic diseases through altered processing of pain circuits and development of psychiatric disorders like depression and anxiety^3,5,6^.

Even though it is known that pain is a very strong physical stressor, hormonal and metabolic changes due to acute or chronic pain have not been studied systematically and in detail so far. In theory, physical reactions to acute or chronic pain should be similar to the physical reactions triggered by acute or chronic stress. In acute stress, the “fight or flight” reaction is triggered, leading to an activation of the sympathicus system, which triggers the activation of the hypothalamo-pituitary-adrenal axis, including secretion of prolactin and vasopressin^7^. As a consequence, lipid and glucose metabolism is changed in order to provide more energy from storage. The gonadal and growth hormone axis and other functions which are not acutely needed, like appetite or digestive function, are depressed. When the acute stressor has passed, metabolism passes into a state of recovery. If stress becomes chronic, adverse metabolic changes of chronic hypercortisolism may appear. Endocrine and metabolic changes have not been consistently reported in acute and chronic pain^8–10^. Furthermore, there exist not enough data to confirm that the endocrine and metabolic pattern of acute and chronic pain is equal to the one of acute and chronic stress^9^. In chronic states of pain, an increased prevalence of the metabolic syndrome and of cardiovascular events has been found^11^, but available data on hormonal axes is inconsistent^8,12–15^. Pain is a subjective sensation; its sensing, conduction, and processing is a complex multidimensional process^16–18^. From a systems perspective, pain generates a complex response that extends beyond the nervous system and contributes to the subjective experience of pain^19^. As a consequence, in the ED, as in other care areas, the current gold standard for pain assessment is the self-rating by patients of their pain intensity using subjective scales^20–22^, like the visual analogue scale (VAS)^23^ or similar tools, which are simple to apply^24^. However, this approach has two obvious limits: these tools remain exclusively subjective, and they require adequate, coherent communication. In a number of patient groups, such as postoperative, critically ill, pediatric, disabled, and geriatric patients, the necessary level of consciousness and communication to assess pain by these scales is not present. This gap is currently partly filled with tools based on behavioral observations^25,26^.

Various methods, focused on an objective quantitative analysis of this perception and pain sensation, have been proposed, included sympathicus system evaluation^20,21,27–32^. Even though these pain intensity assessments have been tested in the experimental setting, they are far from being implemented in clinical use^20,33–35^. As a result, the visual analog scale (VAS) is still used for quantification of pain perception by the patient, while nociception and opioid effects are more often assessed by objective electrophysiological data^36,37^, with inconstant correlation with VAS.

A reliable method to objectively measure pain despite its subjective and multidimensional aspect, be it by blood biomarker or by technical device, has yet to be found or current methods to be improved^38,39^.

States of pain have also been shown to elevate stress biomarkers and inflammatory markers^27,28,40^, and various stress biomarkers as a surrogate marker for pain have been proposed^28,40^, but in clinical practice, there exist few studies^29,41–43^.

Cortisol was one of the first adrenal hormones and stress biomarkers discovered and has remained the prototype of a stress hormone^44^. It has been shown to rise within 30 min after stimulation of experimental pain, but not after local anesthesia of the skin^27^.

The stress biomarker copeptin is the earliest detectable sign of an activated hypothalamo-pituitary-adrenal axis^45,46^. It is a surrogate marker for a sympathetic stimulus^46,47^, and it rises after a physiological stress stimulus^48,49^. It has evolved from a vasopressin precursor to a biomarker for acute coronary syndrome^50^ and for disorders of serum sodium like diabetes insipidus or syndrome of inappropriate secretion of antidiuretic hormone^51–53^. Additional fields of use like prognostic markers in various diseases have been proposed^54^. A rise of copeptin after a stressor like the trier social stress test or exercise has also been reported^48,55^. It has been investigated as a stress biomarker in headache^56^, chest pain^50^ and as a severity marker in acute pancreatitis^57^. In comparison to cortisol, there exists the possibility of point-of-care measurement for copeptin, and for chest pain, it is already implemented in some emergency departments^58^. So far, only one study investigated copeptin in a post-hoc analysis in a hyperalgesia pain model, but without comparison to a placebo group^59^.

We had the opportunity to investigate the potential changes of the stress biomarkers copeptin and cortisol after painful stimulation as an ancillary project in an experimental pain model of electrophysiological stimulation, which tested opioid effects in healthy volunteers (HVs). Even though this experimental secondary analysis was exploratory, we hoped to answer questions which arose from another trial, which evaluated copeptin in a hyperalgesia model^59^. Our primary aim was to investigate whether copeptin will rise after a moderate painful stimulation, similar to cortisol, which was taken as a reference biomarker, and whether such effects would be diminished after systemic opioid administration.

## Methods

### Study design and population

This was a prespecified ancillary analysis of copeptin and cortisol serum concentrations in an experimental pain model in HVs. The main trial was a phase I/II trial, with the aim to compare analgesic effects of inhaled morphine or fentanyl to intravenous administration of morphine in a nociceptive flexion reflex (NFR) model targeting moderate pain. Three parallel treatment arms were tested using a randomized, single-blind, placebo-controlled design. HVs were stratified by sex. The classical “Up and down” methodology^60,61^ was used to determine dose correspondence between inhaled modes versus intravenous mode and to perform a pharmacokinetic analysis^62^. Before randomization the treatment administration, a selection phase was required according to a NFR calibration. The first 21 HVs entering the trial independent of randomization arm also took part in this ancillary analysis.

The study was conducted in accordance with legal and regulatory requirements, as well as the general principles set forth in the International Ethical Guidelines for Biomedical Research Involving Human Subjects (Council for International Organizations of Medical Sciences 2002), Guidelines for Good Clinical Practice (International Conference on Harmonization 1996), and the Declaration of Helsinki (World Medical Association 1996 & 2008). The study protocol and patient-informed consent procedures received regional Ethics Committee approval (Nord-Ouest I, approval number 02/17/2013) prior to registration (reference number 2013/004/HP). Authorization for this protocol was granted by the French Competent Authority-ANSM (art. L 1123-8 of French Public Health Code). Thanks to these two centralized procedures, this study has gained ethical approval at both central and local levels. The study was performed by the Clinical Investigation Center (CIC) of Rouen University Hospital.

This study was registered at clinicaltrials.gov as NCT01975753 on November 5, 2013 before the first subject was recruited. The trial results are reported according to the latest version of the CONSORT Statement^63^. It was performed by the Clinical Investigation Center (CIC) of Rouen Hospital, France.

HVs aged between 18 and 60 were recruited from the CIC's repository of 18–60 year old HVs, or from spontaneous presentation. Before their participation, all HVs were carefully briefed concerning the experimental procedures and signed an informed consent form after a time period for consideration, at the inclusion visit. Exclusion criteria were chronic intake of medication, chronic diseases, active smoking, and impossibility of informed consent. For women, an oral, a mechanical, or a surgical contraception was required due to the use of opioids^64^. HVs on long-term painkillers or psychotropic drugs, or with chronic neuropsychiatric pathologies which may alter the pain threshold, or with active drug history or practice, were also excluded.

For reasons of standardization and reproducibility, HVs presented on the day of assessment at 8 a.m., had fasted for at least four hours, including abstinence from fluids, and without any strenuous exercise for the same period. HVs were free of acute pain, and they were instructed to refrain from alcohol, and from analgesic medication for 48 h prior to testing. After their inclusion, HVs received financial compensation for their participation.

### Hypothesis and endpoints

We hypothesized that copeptin and cortisol would rise after pain induced by electrostimulation, and that this rise would be less when opioids were administered.

Cortisol was taken for proof of the principle, as it has been shown to rise after 30 min in another experimental pain model^27^.

Primary endpoint was change in serum biomarker levels (copeptin and cortisol) after pain stimulation: considering copeptin and cortisol levels, we arbitrarily considered a change of 20% to be significant for each session. For cortisol, a change of at least 20% has been reported after a stimulus^27,55^, while for copeptin, psychologic stress led to an increase by 60% after 20–30 min^55^. For both biomarkers, values were expected to return to baseline values after 60 min^27,55^.

When looking at cortisol profiles during experimental pain, there was a decrease during the resting period before starting the experiment in a previous study^27^. We hypothesized that placing the intravenous line before the experimental model would be a prior pain stimulation, which could be the reason for starting with higher values, which decreased after a period of rest. As, of course, it was not possible to measure a real baseline value before placing the intravenous line, we assumed there would be a fall of biomarker levels between 0 and 10 min, thus during the resting time which preceded the start of the electrophysiological procedures (resting session). For all following statistical calculations, the 10 min biomarker values will be taken as baseline value thereafter.

Prespecified secondary endpoints were correlation between each biomarker levels and pain levels on VAS pain scale, effect of opioids on biomarker levels, and effect of the resting session on biomarker levels.

### Experimental pain model

**Figure 1:**
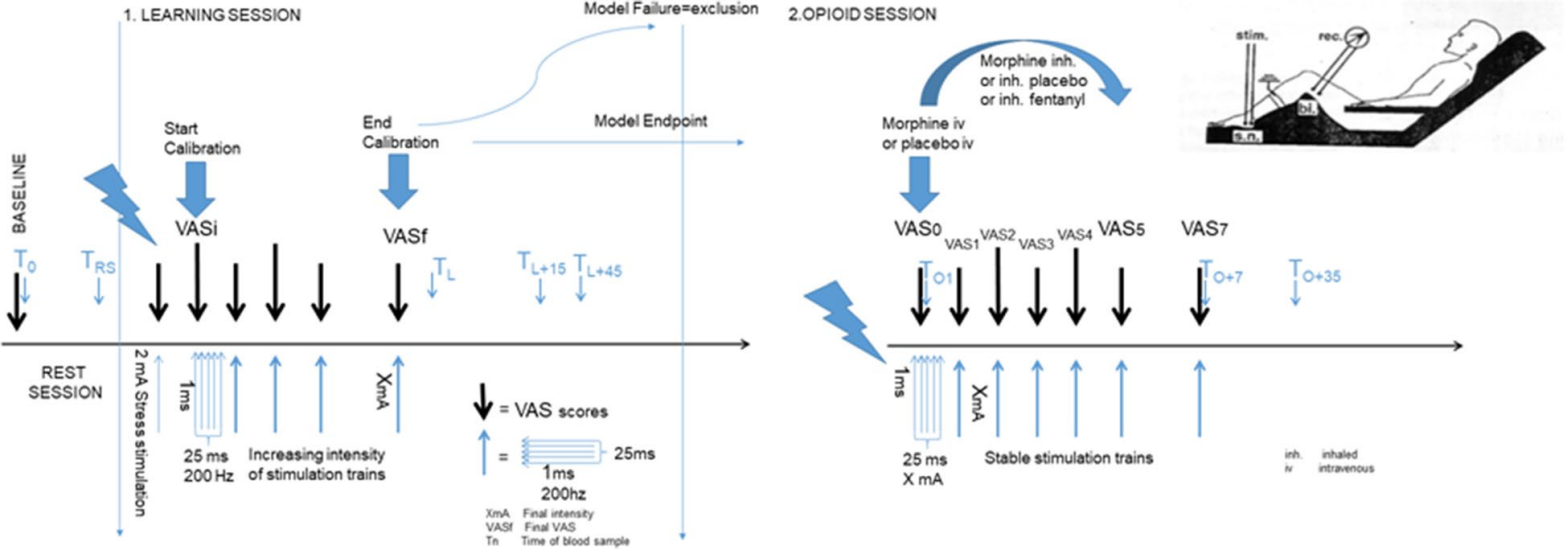
Experimental pain model setup. Adapted from Willer^65^. *VAS* visual analog pain scale rating from 0 to 100, 0 meaning no pain and 100 the worst imaginable pain.

The experimental pain model was based on a NFR model and divided into two consecutive test-sessions on the same day for a 2-h experiment: the learning session, during which electric stimulation trains were calibrated to reach a target pain of VAS between 40–60/100 on pain scale (model endpoint), and the pain treatment session by opioids (opioid session) to evaluate pain relief as VAS ≤ 20/100 or treatment failure (trial endpoint). The NFR model used was adapted from the classical and referenced NFR model^65^ and consisted of electric stimulation trains of the sural nerve (see Fig. 1 for experimental pain model setup). Details are reported elsewhere^62^.

### Procedures

HVs presented at 8 a.m. after an overnight fast (at least 4 h) including abstinence from fluids. After installation of an intravenous line, a resting session constituted of a pause of 10 min. A glass of orange juice was offered prior to the start of the protocol in order to prevent blood glucose variations as recommended in neurostimulation-based experiments^66^. Then, HVs were first introduced and accustomed to the NFR method by pain stimulation with low electrostimulation at 2 mA. Subsequently, HVs underwent the first session of increasing electrophysiological stimulation, until targeted VAS was successfully reached (model endpoint) or not (model failure linked to NFR failure or pain model failure)^65^. Serum blood was drawn in all HVs after installation of the intravenous line, at the end of first trains of electrostimulation, independent of final VAS (L1), at 15 min (L + 15), and at 45 min (L + 45) thereafter (see also Fig. 1).

When the model endpoint was reached (final VAS between 40–60/100), successful HVs underwent the second session of electrophysiological stimulation of stable intensity trains (final intensity), and received at the same time in a double-blinded manner inhaled morphine plus intravenous placebo, inhaled fentanyl and intravenous placebo, or inhaled placebo and intravenous morphine, the opioid session. According to the Dixon method, in each parallel arm of treatment, a minimal dose of opioids was decided at the beginning of the inclusions. Then, increased or decreased doses of opioids were administered to each volunteer, based on the previous VAS pain score and on the success or failure of pain relief. Intravenous morphine was administrated in less than 10 s. A 5-min inhalation was prepared diluting each dose of morphine chlorhydrate (10 mg/mL concentrate) in sodium chloride to a final volume of 3 mL. At a constant airflow rate of 10 L/min, all drug particles were nebulized at the end of the procedure. Morphine doses ranged from 1 to 5 mg and 3–8 mg for intravenous and inhaled routes respectively, with a 0.3 mg increase or decrease. Doses of inhaled fentanyl ranged from 50 to 140 µg, with a 15 µg increase or decrease.

Serum blood was drawn at start of stimulation and administration of opioids (O1), at the trial endpoint time, i.e. 7 min (O + 7), and 35 min thereafter (O + 35).

Systolic blood pressure, heart rate, respiratory rate, oxygen saturation, VAS pain scale, Ramsay score, and biomarkers were measured at baseline, after the resting session, at L1, L + 15, and L + 45 of the learning session, and at O1, O + 7, and O + 35 of the opioid session. Brachypnea, bradycardia, hypotension, and desaturation were stopping criteria for opioid administration (see full algorithm elsewhere)^62^.

### Blood sampling and assays

Blood samples for biomarker measurement were obtained from an indwelling venous catheter. Serum blood was frozen at −80 °C.

Copeptin levels were measured with a highly sensitive TRACE technology assay (COPEPTINEproAVP Kryptor^®^, B.R.A.H.M.S. AG, Hennigsdorf, Germany) on Kryptor Compact Plus with a lower detection limit of the assay of 0.69 pmol/L and a functional assay sensitivity (< 20% interassay CV) of < 1.59 pmol/L)^50,67^.

Serum cortisol was measured using an automated electrochemiluminescence immunoassay (Elecsys Cortisol II, Cobas e602^®^ unit, Roche Diagnostics, Mannheim, Germany) with a measuring range of 1.5 to 1750 nmol/L.

### Sample size considerations and statistical analysis

Based on other copeptin trials^50,56,59,68^, we expected a rise in copeptin levels from around 3 pmol/l to at least 8–12 pmol/l. Using a paired t-test for mean difference, we calculated that, for detecting a difference in copeptin levels of 4 ± 4 pmol/L from the baseline level, with an alpha of 5%, a power of 80% was expected if at least 17 HVs were included.

As a too small sample size may defer non-reproducible results^69,70^, we compared this power calculation to other trials and found similar sample sizes in comparable experimental pain trials^27,55,59^.

All statistical tests were 2-tailed. p < 0.05 was considered significant. Data was analyzed using STATA statistical software (STATA/MP V15.1, STATA Corp., College Station, Tx, USA) and Graph Pad Prism 8.3 (La Jolla, Ca, USA).

We applied a normality test which showed nonparametric distribution. Accordingly, quantitative variables are summarized as medians with their interquartile ranges (IQRs) and qualitative variables as counts (percentage). Paired data and repeated measures data were compared using Wilcoxon matched pairs signed rank test, and unpaired data were compared using Mann–Whitney-U-Test. Multiple group comparison was performed by Friedman’s Test in case of matched or repeated measures data, and by Kruskal–Wallis test in case of unmatched data.

Friedman test was performed to determine an overall difference in biomarker level within the three sessions of the trial (resting session, learning session and opioid session). Wilcoxon matched-pairs test was performed to determine the difference of biomarker levels between baseline and after the resting session as well as between the defined time points of the learning session and opioid session.

Thereafter, a linear regression analysis for delta copeptin and delta cortisol between the resting session and L1 was performed. To detect independent factors for changes in biomarker levels, we evaluated sex, pain intensity as measured by VAS, and pain reaction type. To respect rules to prevent overfitting, we calculated one model for the factor pain reaction type, while the influence of the factors sex and pain as measured by VAS were calculated separately. Linear regression analysis performed similarly for delta copeptin and delta cortisol of the opioid session at O + 7, with the factors as mentioned above.

Furthermore, Spearman rank correlation at the main VAS pain score of the opioid session (at O + 7) between each biomarker levels and VAS pain scores was performed.

To identify difference in biomarker levels between opioid groups (inhaled morphine, intravenous morphine, inhaled fentanyl), a Kruskal–Wallis test was performed for overall differences, and a Mann–Whitney-U-Test to compare biomarker levels between inhaled and intravenous morphine treatment groups.

### Ethics approval

The study protocol and patient-informed consent procedures received regional Ethics Committee approval (Nord-Ouest I, approval number 02/17/2013) prior to registration (reference number 2013/004/HP). Authorization for this protocol was granted by the French Competent Authority-ANSM (art. L 1123-8 of French Public Health Code). Thanks to these two centralized procedures, this study has gained ethical approval at both central and local levels.

### Consent to participate

Before their participation, all HVs were carefully briefed concerning the experimental procedures and signed an informed consent form after a time period for consideration, at the inclusion visit.

## Results

### Baseline characteristics

Of the 45 HVs included in the main trial, 21 completed all sessions and therefore had a complete data and biomarker set available for this analysis. Baseline data of the included HVs are shown in Table 1, and detailed biomarker levels at each timepoint are shown in Table 2.

**Table 1:** Characteristics of subjects and of nociceptive reflex.

	n = 21
Age, years	28 (IQR 25–35)
Female sex, n (%)	11 (52%)
**Anticonception**	
Estrogen progestin pill	5 (24%)
Estrogen progestin vaginal ring	1 (5%)
Progestin implanon	1 (5%)
Copper contraceptive coil	1 (5%)
Menopause	3 (14%)
Body mass index (kg/m^2^)	23.9 ± 2.1
Heart rate per minute	68 ± 11
Systolic blood pressure, mmHg	120 ± 14
Morphine aerosol, n (%)	9 (43%)
Fentanyl aerosol, n (%)	3 (14%)
Intravenous morphine, n (%)	9 (43%)
Baseline cortisol (nmol/L)	458 ± 267
Baseline copeptin (pmol/L)	5.1 ± 2.7
Stimulation time of LS, min	19 (IQR 12.5–25.5)
Intensity of stimulation during LS, mA	22 ± 11
VAS at end of LS	47 ± 6
Stimulation time of OS, minutes	2 (IQR 0.75–5.0)
Intensity of stimulation during OS, mA	25 ± 12
VAS at O + 7	52 ± 1.7

n = 21 includes only subjects who completed all test sessions and had a complete data set available.

Normally distributed data is shown as mean ± SD, otherwise as median (IQR).

*LS* learning session, *OS* opioid session, *VAS* visual analog pain scale rating from 0 to 100, 0 meaning no pain and 100 the worst imaginable pain.

**Table 2:** Biomarker levels at each timepoint.

	Cortisol (nmol/L)	Copeptin (pmol/L)
Baseline	366 (321–493)	4.553 (2.665–6.456)
Baseline after resting session	321 (279–483)	4.496 (3.001–6.409)
L1: time point when reaching target pain	318 (259–498)	4.036 (2.27–5.332)
L + 15: time point L1 plus 15 min	295 (212–437)	3.617 (2.145–4.805)
L + 45: time point L1 plus 45 min	255 (206–392)	3.678 (2.399–4.535)
O1: time point of administration of opioids in addition to target pain	211 (162–308)	3.683 (2.721–4.534)
O + 7: 7 min after O1	246 (191–316)	3.594 (2.618–4.499)
O + 35: 35 min after O1	221 (142–268)	3.439 (2.375–4.34)

n = 21 includes only subjects who completed all test sessions and had a complete data set available.

Data is shown as median (IQR).

### Cortisol

**Figure 2:**
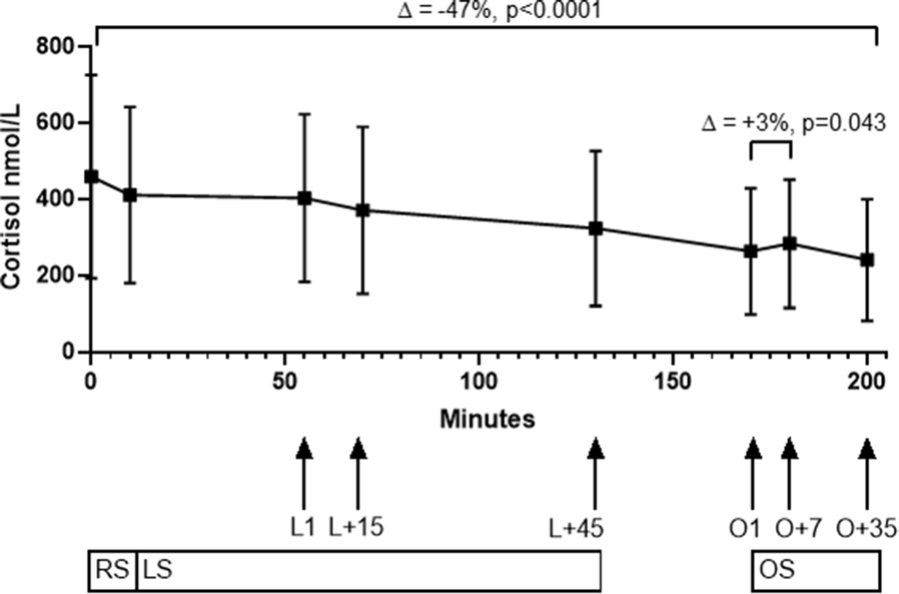
Time plot of cortisol during all test sessions. *RS* resting session. ∆ = −13%, p < 0.0001. *LS* learning session. ∆ = −24%, p = 0.0002. *L1* time point when reaching target pain. *L + 15* time point L1 plus 15 min. *L + 45* time point L1 plus 45 min. *OS* opioid session. ∆ = −18%, p = 0.0491. *O1* time point of administration of opioids in addition to target pain. *O + 7* 7 min after O1. *O + 35* 35 min after O1. Arrows point to the corresponding time points. Boxes and whiskers represent mean and SD. Spearman rank correlation between cortisol levels and VAS scores at the trial endpoint time showed no correlation between VAS scores and cortisol levels (Spearman r −0.42 (95% CI −0.74 to 0.055), p = 0.07).

Friedman test showed an overall difference of cortisol throughout the whole test time (mean difference −195 nmol/l (−273 to −135 nmol/l), −47%, p < 0.0001), also represented in Fig. 2 (cortisol time plot) and Supplemental Fig. 1.

A significant decrease of cortisol during the resting session was observed (median difference −44 nmol/l (−65 to −26 nmol/l), −13%, p < 0.0001).

Within the learning and opioid sessions, this overall decrease of cortisol levels was confirmed. During the learning session, the median difference was −83 nmol/l (−44 to −124 nmol/l) or −24% (p = 0.0002). During the opioid session (O1 to O + 35), the median difference was – 34 nmol/l (−56 to 9 nmol/l) or −18% (p = 0.0491). However, between O1 to O + 7, the median difference was + 8 nmol/l (−9 to 66 nmol/l) or + 3% (p = 0.043). When looking at individual data, 9 subjects had a drop and 11 subjects had an increase in cortisol values (see also Supplemental Fig. 1).

Linear regression analysis did not show an influence of pain reaction type on cortisol values, neither in the learning session nor in the opioid session. While pain measured by VAS did not influence cortisol levels in neither session, male sex was associated with a higher cortisol value in the opioids session (Coefficient 38.7, 95% CI 2.3–75, p = 0.039), but not in the learning session (Coefficient − 27.7, 95%CI −06 to 40, p = 0.4).

Considering groups of opioid administration, i.e. inhaled or intravenous morphine or intravenous fentanyl, no difference between cortisol levels was identified by Kruskal–Wallis-Test for multiple comparisons (p = 0.85).

### Copeptin

**Figure 3:**
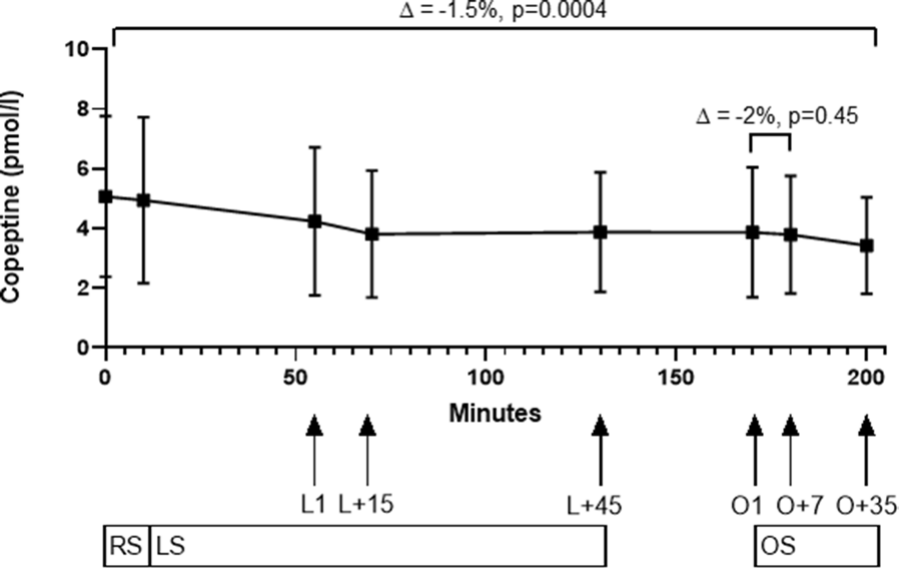
Time plot of copeptin during all test sessions. *RS* resting session. ∆ = −0.9%, p = 0.14. *LS* learning session. ∆ = −22%, p = 0.0003. *L1* time point when reaching target pain. *L + 15* time point L1 plus 15 min. *L + 45* time point L1 plus 45 min. *OS* opioid session. ∆ = −6%, p = 0.1. *O1* time point of administration of opioids in addition to target pain. *O + 7* 7 min after O1. *O + 35* 35 min after O1. Arrows point to the corresponding time points. Boxes and whiskers represent mean and SD. Spearman rank correlation between copeptin levels and VAS scores at the trial endpoint time showed no correlation between VAS scores and copeptin levels (Spearman r − 0.26 (95% CI −0.65 to 0.25), p = 0.31).

Friedman test showed an overall difference in copeptin levels throughout the whole study (median difference −1.02 pmol/l (−0.3 to −0.02 pmol/l), −1.5%, p = 0.0004). Time plot of copeptin levels are represented in Fig. 3 and Supplemental Fig. 2. During the resting session, no significant decrease was observed (median difference − 0.05 pmol/l, −0.9%, p = 0.14).

The main decrease of copeptin levels occurred during the learning session (median difference −1.01 pmol/l (−2.35 to 0.08 pmol/l), −22%, p = 0.0003), while in the opioid session, copeptin levels were stable (median −0.22 pmol/l (−0.69 to 0.09), − 6%, p = 0.10). There was no visible peak at O + 7 (median change −0.003 pmol/l (−0.50 to 0.24), −2%, p = 0.45). Linear regression analysis did not show an influence of pain reaction type on copeptin values, neither in the learning session nor in the opioid session. While pain measured by VAS did not influence copeptin values, neither in the learning session nor in the opioid session, male sex was associated with a lower delta copeptin in the learning session (Coefficient −0.63, 95%CI −1.13 to −0.13, p = 0.017), but not in the opioid session (Coefficient −0.30, 95%CI −0.90 to 0.30, p = 0.31).

Considering groups of opioid administration, i.e. inhaled or intravenous morphine or intravenous fentanyl, no difference between copeptin levels was identified by Kruskal–Wallis-test for multiple comparisons (p = 0.311).

As there were seven women using a hormonal anticonception method, which potentially may have an influence on serum cortisol levels and on hormonal stress response^71^, we did a sensitivity analysis excluding these seven women. Neither the overall test for cortisol nor the within-session results differed from the whole cohort in the sensitivity analysis. Furthermore, the median increase of cortisol between O1 and O + 7 was again shown with a median difference + 7.5 nmol/l (p = 0.0047).

## Discussion

In this study investigating the effects of different opioids in a NFR pain model targeting moderate pain, we showed that cortisol decreased throughout the sessions by 43% despite pain stimulation, except shortly after administration of opioids, at which time point it increased by 3%. Copeptin decreased throughout the study by 30%, mainly until the end of the learning session, but not during the opioid session.

There was no difference of biomarker levels between randomized groups of opioids.

Looking for a reason for decreasing cortisol and copeptin levels during the test, the main and apparent reason was probably circadian rhythm. For cortisol, such an effect is well known, potentially accounting for a 30–50% decrease between 07h00 and 12h00^72–74^. Our trial started at 09h00 and ended in most HVs at 12h00. Circadian rhythm has been observed as a strong confounding factor for cortisol changes previously^75^. For Copeptin, the effect of circadian rhythm has been reported to be much smaller^72,76^, and it has so far not been identified to be a relevant confounder. An additional plausible influencing factor was sex. While delta cortisol was associated in men in the opioids session, this is not supported by previous research, which generally showed lower cortisol values in both men and women after opioid administration^77^. Therefore, this finding may be due to chance. Male sex was also associated with a lower delta copeptin in the learning session. This finding is supported by data from Kacheva et al., which have shown an association of stress-related copeptin increase in women but not in men^68^.

As measurement of pain anticipation is limited, we accounted for pain anticipation by performing measurements during the resting session, showing a significant decrease of cortisol of 12%, while the decrease was only 5% for copeptin. For both cortisol and copeptin, an increase after psychologic stress testing has been shown^55^. Therefore, in addition to considerations linked to circadian rhythm, an initially high biomarker level due to anticipation must be expected.

Even though the time points for biomarker measurement were slightly different between learning session and opioids session, we believe this cannot be the main reason for the non-detection of an increase in the learning session. Due to the original setup of the experiment, it was not possible to strictly synchronize the time points.

One of the plausible hypotheses was rather that the initial placement of the intravenous line can already lead to an increase of stress biomarkers. However, this trial was not able to demonstrate such an effect, as there was no significant decrease of either copeptin or cortisol during the resting session at a HV population level. For cortisol, this stands in contrast to another study, which measured saliva cortisol^78^, but results of a systematic review on this matter were inconclusive, revealing that about 1/3 of studied subjects displayed an increase of either serum or saliva cortisol after venipuncture^53^. For copeptin, this has not been studied before. However, when looking at individual biomarker values, in our trial, 18 out of 21 HVs had a decrease between the first measurement and the resting session. As this was an ancillary project, it was not possible to adapt the original study setup. It is also possible that a slightly longer resting period of 30 min would have shown a decrease of 20% of biomarker values, but we believe it to be negligeable, as the painful stimulation following the resting period did not lead to a significant increase in biomarker levels, either.

Otherwise, we report a small cortisol peak at O + 7 of the opioid session, but no such increase was visible in the learning session at L + 15. Copeptin did not show a veritable peak at the same time point, but it did not decrease either, which could be interpreted as a small rise, too. We interpreted this rise in biomarkers as an opioid effect and not as a pain effect for the following reasons: first, pain exposure was much longer in the learning session due to pain threshold determination based on a slow and continuously increasing pain intensity until a VAS of 40–60 was reached. Second, no tolerance phenomenon was observed; third, the main difference between the learning session and the opioid session was opioid administration. Data on cortisol release after opioid administration is controversial, but not in line with our results. While ACTH and cortisol subsequently seem to increase after acute pain and acute opioid administration in the animal model, cortisol levels seem to decrease in humans^79^. So far, data on the effect of opioids on arginine vasopressin have shown controversial results^79^. A recent study has shown an increase of copeptin levels associated with fentanyl administration^59^, supporting our interpretation, even though the effect we observed was quite small.

This experimental protocol, investigating the pharmacologic titration of opioids, required the subjects to come in fasting for at least 4 h, which included not drinking fluids. This is in general not particularly favorable for the measurement of copeptin, as it may cause copeptin levels to rise. To counteract effects of fluid and food restriction, subjects were given a glass of orange juice before starting the trial procedures. Even though these preventive four hours of abstinence of fluid intake may not seem a relevant issue, the fluid abstinence was continued throughout the test for another 4 h. In comparison, a water deprivation test used to distinguish primary polydipsia from diabetes insipidus takes between five and eight hours, depending on the protocol^52^. While copeptin values are reported to be much higher in HVs than in patients with primary polydipsia or even diabetes insipidus^52^, median copeptin in HVs was about 4 pmol/l after overnight fluid restriction in one trial, but continued to rise to about 12 pmol/l during the following eight hours of water deprivation^80^. In another protocol investigating water deprivation in HVs, an increase of copeptin levels was shown after 24 and 28 h of fluid restriction^81^. Certainly, the fact that in our cohort the HVs had to refrain from drinking water at least four hours before the experimental trial, presents a potential confounder for the baseline copeptin value. The offered glass of orange juice might have counteracted any adverse effects of fluid restriction as desired.

Kirschbaum et al. have shown that ready availability of energy is a prerequisite for a rise in cortisol due to the trier social stress test^82^. In subjects fasting before undergoing the trier social stress test, the rise in cortisol was blunted compared to subjects receiving glucose or water before the test. As our subjects received a glass of orange juice, we consider this factor to be negligible in our trial.

Overall, our results do not support results of another study investigating biomarkers in a similar experimental pain model. Greisen et al. showed an increase of cortisol, epinephrine and norepinephrine between 20 and 60 min after painful stimulation^27^. However, they measured biomarkers every 10 min after pain stimulation, whereas we only measured them at 15 min and 45 min after pain stimulation in the learning session, and at 8 min and 35 min during the opioid session. At 8 or 15 min after painful stimulation, a peak would potentially have been visible in the Greisen study, but at 45 min, when epinephrine and norepinephrine peaks were already abated, a peak would probably still have been visible for cortisol. The main issue could be that the Greisen study induced pain with goal of VAS of 80/100, which is not moderate pain, as specifically targeted in our study.

In summary, the hypothesis that acute pain will lead to somatic stress with a systemic reaction strong enough for a rise in serum copeptin and/or cortisol levels is not supported by our experimental data. External factors, especially circadian variation, pain anticipation, or opioid effects are much stronger factors associated with changes in cortisol or copeptin in this cohort of HVs exposed to moderate pain in an NFR model. Therefore, copeptin and cortisol are not useful biomarkers as a surrogate for acute moderate pain. As a second consequence, it may not be necessary to consider acute moderate pain as a confounding factor for cortisol or copeptin levels in the clinical setting.

### Limitations

There are several limitations to this study. First, this induced pain model aimed to reach a moderate pain, defined by a VAS between 40 and 60. A stronger pain stimulus might have induced a small peak in biomarker levels.

Second, the timing of the entire experiment starting at 8 a.m. has introduced the bias of circadian rhythm of biomarker levels, which has had a major influence on the levels of cortisol, and to a smaller extent of those of copeptin.

Third, the fact that the subjects had to come in fasting and without intake of fluids for at least four hours might have led to higher baseline copeptin levels than reported in other trials who did not restrict fluid intake prior to the start of the trial. Nevertheless, the offered glass of orange juice before the learning session may have counteracted this effect.

Fourth, timing of blood sampling may have led to false negative results by missing a potential decrease after the resting session and accordingly, an increase of biomarkers 30 min after the start of painful stimulation. Furthermore, the timing of blood sampling between the learning session and the opioids session were slightly different, due to the original setup of the experiment.

Fifth, even though HVs were carefully selected, residual hyperalgesic pain or chronicity cannot be ruled out as potential influencing factors.

Sixth, even though sample size was larger than other cohorts, the population of the study remains small, especially for subgroup analyses according to different opioids and routes of administration.

## Conclusion

In this experimental pain model in healthy volunteers performed in the morning and targeting moderate VAS pain scores by electrostimulation , cortisol levels were lower after 10 min of rest than at baseline, indicating initially 12% higher levels probably due to pain anticipation, whereas change in copeptin levels was inconclusive. Neither cortisol nor copeptin levels increased after moderate pain according to VAS, whereas we observed a small cortisol peak after administration of opioids. However, main induced changes were much lower than circadian variation or pain anticipation, indicating a negligible relevance for clinical practice.

## Supplementary Information

Supplementary Figure S1.

## Data Availability

The datasets generated analysed during the current study are available from the last author V.E.L. on reasonable request.

## Abbreviations

ANSM: Agence Nationale de Sécurité du Medicament et des Produits de Santé (French Competent Authority as by art. L 1123-8 of French Public Health Code)
CIC: Clinical Investigation Center
HVs: Healthy volunteers
L1: Time point when expected target pain reached at the end of the learning session
L + 15: Time point L1 plus 15 min
L + 45: Time point L1 plus 45 min
NFR: Nociceptive flexion reflex
O1: Time point of beginning of opioid administration in the opioid session
O + 7: 7 minutes after O1
O + 35: 35 minutes after O1
VAS: Visual analog scale
