# Supplementary material for: Relevance of cortisol and copeptin blood concentration changes in an experimental pain model
Source: Sci Rep. 2022 Mar 19;12:4767. doi: 10.1038/s41598-022-08657-4 (PMC8934351; doi:10.1038/s41598-022-08657-4)
Supplement: Supplementary file 1 — Supplementary Figure S1. [file 41598_2022_8657_MOESM1_ESM.pdf]

SUPPLEMENTARY INFORMATION

To

Relevance of cortisol and copeptin blood concentration changes in an experimental pain model

Claudine A. Blum, Laëtitia Velly, Christine Brochet, Frédéric Ziegler, Marie-Pierre Tavolacci, Pierre Hausfater, Virginie Eve Lvovschi

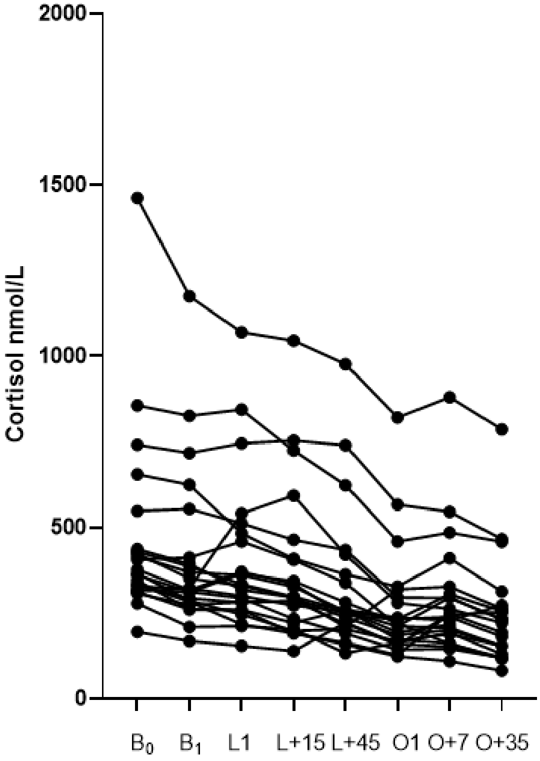

Supplemental Figure 1. Individual cortisol values at each measured time point.

B_0: Baseline measurement

B_1: Baseline measurement after 10 minutes rest

L1: Time point when reaching target pain

L+15: Time point L1 plus 15 minutes

L+45: Time point L1 plus 45 minutes

O1: Time point of administration of opioids in addition to target pain

O+7: 7 minutes after O1

O+35: 35 minutes after O1

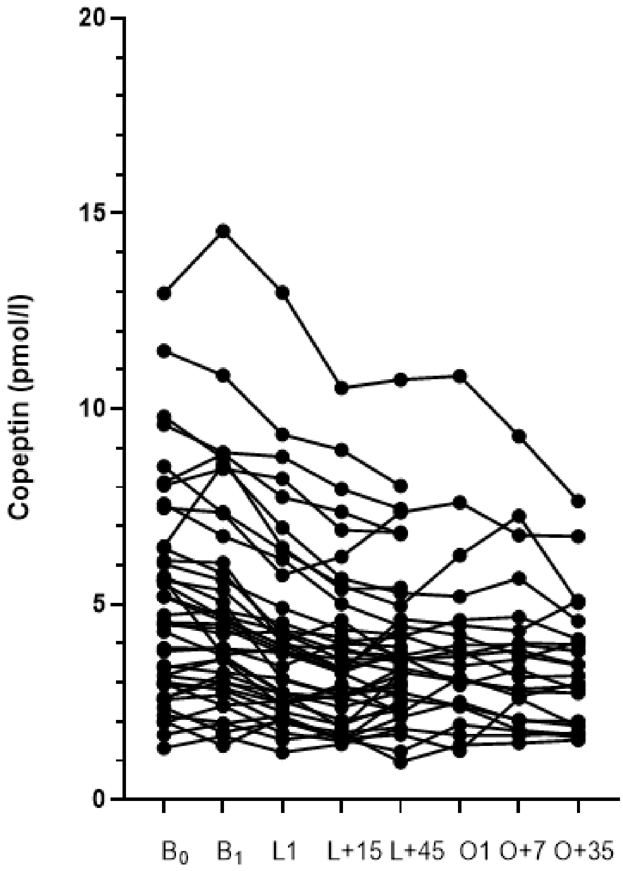

Supplemental Figure 1. Individual copeptin values at each measured time point.

B_0: Baseline measurement

B_1: Baseline measurement after 10 minutes rest

L1: Time point when reaching target pain

L+15: Time point L1 plus 15 minutes

L+45: Time point L1 plus 45 minutes

O1: Time point of administration of opioids in addition to target pain

O+7: 7 minutes after O1

O+35: 35 minutes after O1
